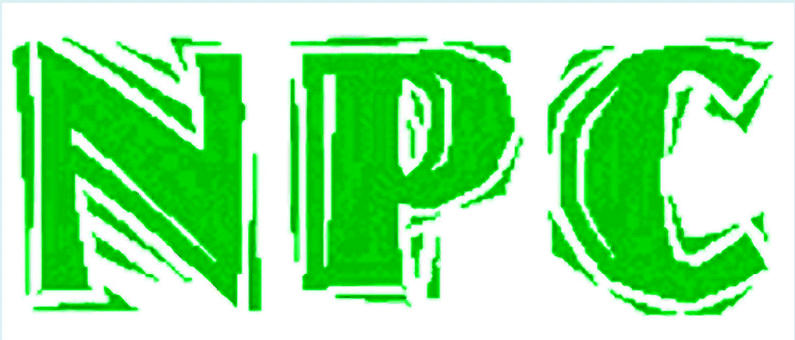# EHPnet: Noise Pollution Clearinghouse

**Published:** 2005-01

**Authors:** Erin E. Dooley

Not all sound is bad, but too much of the wrong sounds harm your health. What many people don’t know is that everyday items such as lawn mowers and kitchen blenders can emit noise at hazardous levels. More than 30 million Americans work at al job where they are exposed to hazardous sound levels on a regular basis. One-third of Americans with some degree of hearing loss can attribute that loss to sound exposure. And the evidence is building to point toward other noise-related health effects. The Noise Pollution Clearinghouse (NPC) is one group whose mission is to foster awareness of noise-related issues. On the NPC’s website, located at **http://www.nonoise.org/**, visitors can find many different resources to learn about what noise pollution is and how it can be fought.

The NPC has four ongoing campaigns: Quiet Classrooms, Quiet Lawns, Quiet Lakes, and Silencing Car Alarms. The Quiet Classrooms portion of the site offers tips to students, teachers, and others on how to make the learning environment as quiet as possible, while the Quiet Lawns page rates 40 different lawn mowers in terms of noisiness. The Quiet Lakes page features information on the noise caused by sport watercraft and what the NPC is doing to fight this noise source. The Silencing Car Alarms portion of the site tells why the NPC thinks car alarms should be outlawed and lists quieter alternatives for keeping cars safe from thieves.

For the layperson, the NPC has assembled an online library of almost 50 articles, reports, and seminal documents from a variety of sources. Within this section is a dictionary of noise terms, a primer on environmental noise, and more technical documents from national and international experts. A separate library contains noise-related documents just from the U.S. Environmental Protection Agency. This page also links to the full text of the Noise Control Act and federal regulations from the Office of Noise Abatement and Control, as well as to *Noise Effects Handbook: A Desk Reference to Health and Welfare Effects of Noise*. This 10-chapter textbook was written by the Office of Noise Abatement and Control to address effects ranging from fetal impacts to how loss of hearing affects speech and other activities.

Starting once more from the homepage, the Hearing Loss and Occupational Noise Library includes documents from the Occupational Health and Safety Administration, the National Institute for Occupational Safety and Health, and the Mining Safety and Health Administration. Located here are criteria, guides, and standards for protecting workers’ hearing, plus a bibliography of 2,500 references on hearing and ear protection, among other topics. The NPC also is building an online noise law library with federal, state, local, and European noise-related laws and regulations as well as proposed regulations.

For people who want to put their knowledge to work, the NPC Resource Library page has links for activists, educational resources, upcoming noise conferences and meetings, and potential funding sources. The NPC also provides pages on its website for local noise organizations that could not otherwise afford to host their own sites.

## Figures and Tables

**Figure:**